# Role of Heat Shock Proteins in Atrial Fibrillation: From Molecular Mechanisms to Diagnostic and Therapeutic Opportunities

**DOI:** 10.3390/cells12010151

**Published:** 2022-12-30

**Authors:** Daiqi Liu, Xuyao Han, Zhiwei Zhang, Gary Tse, Qingmiao Shao, Tong Liu

**Affiliations:** 1 Tianjin Key Laboratory of Ionic-Molecular Function of Cardiovascular Disease, Department of Cardiology, Tianjin Institute of Cardiology, Second Hospital of Tianjin Medical University, Tianjin 300211, China; 2 Cardiac Electrophysiology Unit, Cardiovascular Analytics Group, Hong Kong, China; 3 Kent and Medway Medical School, Canterbury CT2 7NZ, UK

**Keywords:** heat shock proteins, atrial fibrillation, anti-arrhythmic treatment

## Abstract

Heat shock proteins (HSPs) are endogenous protective proteins and biomarkers of cell stress response, of which examples are HSP70, HSP60, HSP90, and small HSPs (HSPB). HSPs protect cells and organs, especially the cardiovascular system, against harmful and cytotoxic conditions. More recent attention has focused on the roles of HSPs in the irreversible remodeling of atrial fibrillation (AF), which is the most common arrhythmia in clinical practice and a significant contributor to mortality. In this review, we investigated the relationship between HSPs and atrial remodeling mechanisms in AF. PubMed was searched for studies using the terms “Heat Shock Proteins” and “Atrial Fibrillation” and their relevant abbreviations up to 10 July 2022. The results showed that HSPs have cytoprotective roles in atrial cardiomyocytes during AF by promoting reverse electrical and structural remodeling. Heat shock response (HSR) exhaustion, followed by low levels of HSPs, causes proteostasis derailment in cardiomyocytes, which is the basis of AF. Furthermore, potential implications of HSPs in the management of AF are discussed in detail. HSPs represent reliable biomarkers for predicting and staging AF. HSP inducers may serve as novel therapeutic modalities in postoperative AF. HSP induction, either by geranylgeranylacetone (GGA) or by other compounds presently in development, may therefore be an interesting new approach for upstream therapy for AF, a strategy that aims to prevent AF whilst minimizing the ventricular proarrhythmic risks of traditional anti-arrhythmic agents.

## 1. Introduction

Atrial fibrillation (AF) is associated with a four- to five-fold increased risk of stroke, a two- to three-fold increased risk of heart failure, and a roughly two-fold increased risk of overall mortality [1,2]. It is estimated that by 2050 AF will be diagnosed in at least 10 million in the United States [3] and 72 million individuals in Asia [4]. The number of patients with AF in the European Union was 9 million in 2010, and this figure is anticipated to double by 2060 due to the rapid aging of the population [5]. AF is an age-related disease associated with congestive heart disease and cardiovascular disease [6]. It results from electrophysiological disturbances that are associated with molecular changes in the cardiomyocytes [7]. In metabolic diseases such as hyperthyroidism or hypothyroidism and diabetes, the incidence of AF also increases with degeneration of the myocardium. Despite the fact that the clinical condition of AF is widely documented, its underlying causes and triggers have not yet been identified.

Heat shock proteins (HSPs) are endogenous protective proteins and biomarkers of cell stress response that mainly include HSP70, HSP60, HSP90, and small HSPs (HSPB). HSPs were first discovered to operate as cell protectors when exposed to high temperatures. Following this discovery, researchers discovered that HSPs also function as molecular chaperones, which are essential for protein folding, intracellular protein transport, and the reaction to unfolded and denatured proteins brought on by heat and other stresses. Emerging research suggests that AF structural remodeling may be caused by disrupted proteostasis in cardiomyocytes, which involves controlling the concentration, conformation, binding interaction, kinetics, and location of individual proteins [8]. Cells respond to a loss of proteostatic control by inducing a heat shock response (HSR), upon which HSPs are expressed. These HSPs are considered to constitute the first line of defense against the proteostasis derailment of cells by stabilizing the sarcomere. In the early stages of AF, HSPs are activated by a stress and illness state through the activation of heat shock transcription factor 1 (HSF1). The small HSPs (HSPB) appear to play a significant role in cardiomyocyte proteostasis by stabilizing contractile proteins and inhibiting degradation [9].

In this review, we aim to provide a concise overview of the current evidence regarding the role of HSPs in atrial remodeling processes as well as in the development and maintenance of AF. Importantly, the function of HSPs and their interacting proteins in AF are discussed. Understanding the significance of protein–protein interactions (PPIs) between HSPs and other proteins in AF will provide an insight into how to therapeutically assist HSPs in the treatment of AF. The potential clinical consequences of HSPs in the therapy of AF are also highlighted.

## 2. Definition and Function of HSPs in AF

Heat shock proteins (HSPs) are a group of chaperone proteins produced by both unicellular and pluricellular organisms in response to different categories of stress conditions. The HSP family ranges in molecular size from 10 to 150 kDa and can be found in all principal cellular compartments. The majority of studies characterized the HSP family based on their molecular weight, mostly focusing on small HSPs (HSPB), HSP60, HSP70, HSP90, and HSP110. They act as a network to maintain proteostasis in cells as well as an efficient first line of defense in response to stress. HSPs were initially described by Ferruccio Ritossa in *Drosophila melanogaster* in the 1960s, but it was not until the 1980s that William Currie explored heart tissue in depth [10,11].

Under pathological circumstances, the HSP family can be responsive to damage induced by temperature, poisons, hypoxia, infectious agents, radiation, and other stresses. In general, HSPs act as molecular chaperones to regulate protein folding, localization, degradation and function, thereby maintaining proteostasis and preventing various forms of cardiomyocyte damage [12]. Numerous systemic disorders are caused by cellular proteostasis derailment, including cardiovascular disorders [13]. The heat shock response (HSR) is triggered by the loss of proteostatic control in cells, which results in the expression of HSPs. Additionally, heat shock factor 1 (HSF1) is one of the central regulators of the HSR. As soon as it is activated, this evolutionarily conserved transcription factor binds to heat-shock-responsive DNA elements (HSEs) and upregulates the genes that encode HSPs [14].

Clinical data indicate that exhaustion of the HSPs is the basis of AF. It has been established that AF-induced proteostasis derailment and consequent electropathological remodeling are caused by abnormalities in the HSR. In individuals with paroxysmal AF, increased HSP27 expression may protect myocytes against myolysis and prevent the development of persistent AF. Higher atrial HSP27 levels are related to a short-duration of AF and a lesser extent of structural damage. Additionally, with progression to more persistent AF stages, the HSP27 levels become exhausted [9]. In line with this, HSP70 levels in atrial tissue were observed to have an inverse relationship with the occurrence of postoperative AF in patients undergoing coronary artery bypass surgery (CABG) [15]. In two other studies, the mitochondrial heat shock proteins HSP60 and HSP10 were found to be overexpressed in atrial tissue from AF patients. These HSPs may play a protective role by preserving mitochondrial integrity and adenosine triphosphate (ATP) production capacity [16,17]. Moreover, the restoration of sinus rhythm after mitral valve surgery in patients with persistent AF is associated with HSF1 activity and induced HSP27 levels [18]. Accordingly, the HSR is active in short-duration AF while it gradually diminishes in persistent AF. HSR exhaustion, followed by low levels of HSPs, can accelerate structural damage to cardiomyocytes, resulting in long-standing and permanent AF.

## 3. Protein–Protein Interactions (PPIs) of HSP Complexes in Atrial Fibrillation

Protein–protein interactions (PPIs) play an important role in cellular biochemical events, regulating biological information and directing cell destiny. Understanding the pathogenic process of human illnesses is aided by research into the involvement of PPIs. HSPs can interact with numerous proteins to conduct a number of biological roles in heart disease, such as autophagy, inflammation, endoplasmic reticulum stress, and oxidative stress.

### 3.1. HSP60 and Atrial Fibrillation

HSP60 is a mitochondrial protein that also exists in the cytosol, endoplasmic reticulum, and plasma membrane. The function of HSP60 is mostly determined by its distribution. HSP60 and HSP10 are required proteins for the proper function and homeostasis of mitochondrial proteins. HSP60 regulates protein folding and prevents protein aggregation in the mitochondria. HSP10, a mitochondrial chaperone, aids in the folding and assembly of proteins transported into the mitochondrial matrix [19]. In cardiomyocytes, high levels of HSP60 and HSP10 exert protective effects on electron transport chain (ETC) complexes. Complexes III and IV are specifically elevated in cardiomyocytes overexpressing HSP60 or HSP60/10. In mice, HSP60 deletion leads to decreased mitochondrial complex activity, decreased mitochondrial membrane potential (ΔΨmito), and increased reactive oxygen species (ROS) generation [20]. Additionally, intracellular overexpression of HSP60 protects cardiac myocytes from apoptosis by blunting the release of cytochrome c and the activation of caspase-3 [19]. This antiapoptotic effect of HSP60 may be in part because HSP60 binds to Bax (B-cell lymphoma-2 associated X) and prevents its translocation into mitochondria, whereas the complex is destroyed under hypoxia, resulting in mitochondrial dysfunction [21,22]. Instead of atrial myocytes, ventricular cardiomyocytes or hypoxia-reoxygenation cardiac myocyte models (hypoxia for 6 hours in an anaerobic workstation) were utilized in these studies. The precise mechanism by which HSP60 protects against AF is unknown. As fully discussed in Section 4.1 below, the researchers use the HSP60/10 characteristics to predict the onset of AF and the stabilization of sinus rhythm after undergoing mitral valve surgery.

Conversely, extracellular HSP60, as a ligand of Toll-like receptor 4 (TLR4), induces myocyte apoptosis through the TLR4–MYD88–p38/nuclear factor kappa-B (NF-κB) pathway, which may increase AF burden [23]. Both tachypaced atrial HL-1 cardiomyocytes and left atrial appendages (LAAs) of paroxysmal or persistent AF patients display mitochondrial stress, as evidenced by increased HSP60 and HSP10 expression, decreased ATP production, a loss of the mitochondrial membrane potential, and mitochondrial network fragmentation, resulting in contractile dysfunction and AF progression [24].

### 3.2. HSP70 Family and Atrial Fibrillation

The HSP70 family (HSP70s) (size range: 70−78 kDa) is classified into two groups: constitutive members (HSC70, HSP75, HSPA5 (glucose-regulated protein 78 [GRP78] and HSPA9 [GRP75])) and inducible members (HSP70), all of which are ATP dependent. HSP70s are expressed in different cellular locations, including the cytosol, nucleus, endoplasmic reticulum (ER), and mitochondria, and are secreted. Their main functions are to maintain the dynamic balance of the synthesis, folding, degradation, and translocation of proteins.

Recent research has revealed that ER-stress (ERS) and the subsequent activation of macroautophagy (hereafter autophagy) play an important role in the onset and maintenance of AF [25,26]. The ERS-induced activation of autophagy is an essential mechanism of atrial remodeling in AF. Autophagy is an evolutionarily conserved protein-breakdown mechanism that sequesters damaged or expired proteins and organelles into autophagosomes for eventual lysosomal degradation. While autophagy is required for normal physiological function, excessive autophagy is harmful. Brundel, B.J. and colleagues developed experimental cardiomyocyte and dog models that mimic persistent AF in patients and exhibit reversible electrical and irreversible structural remodeling. HL-1 atrial cardiomyocytes were treated with 6 Hz tachypacing (1 Hz for normal pacing) to induce a frequency rise with tachypacing (six-fold increase) comparable to what happens during human AF. Tachypacing-induced contractile dysfunction of HL-1 cardiomyocytes, and the ER-stress response, can activate the autophagy–lysosome pathway via the unfolded protein response (UPR) [26]. The UPR stimulates the phosphorylation of the α-subunit of eukaryotic initiation factor 2 (eIF2) at serine 31, which inhibits protein translation and initiates the selective expression of stress-responsive genes such as activating transcription factors (ATF) 4 and 6. ATF4 and ATF6 signaling, in turn, increase the expression and activation of the CCAAT/enhancer-binding protein (C/EBP) homology protein (also known as CHOP) and various autophagy proteins, such as autophagy related 12 (ATG12), MAP1LC3B (also known as LC3) and BiP (also known as GRP78), which together stimulate autophagosome elongation and autophagic protein degradation [14,27]. ER-stress inhibition with the pharmacological chaperone 4-phenylbutyrate (4-PBA), overexpression of the ER chaperone GRP78, or mutation of the eIF2 gene could limit autophagy and thus prevent electrical and contractile failure in AF models [26]. The aforementioned results were also confirmed in a dog model that underwent a 7-day atrial tachycardia stimulus at a rate of 10 Hz to stimulate AF-related atrial remodeling [26]. Liu et al. investigated the role of GRP75 (HSPA9) in the modulation of ER-stress in low-dose streptozotocin induced type 2 diabetes (T2DM)-related atrial remodeling in rats. The contact points between the ER and the mitochondria are known as mitochondria-associated ER membranes (MAM). Calcium can be released from the ER via intracellular calcium release channels (IP3Rs) and voltage-dependent anion channels (VDACs) at the outer mitochondrial membrane (OMM), finally moving to the mitochondria. In diabetic atrial remodeling, the IP3R1-GRP75-VDAC1 complex mediates ERS-MAM-mitochondrial oxidative stress and plays an important role in diabetic atrial remodeling. A higher percentage of AF was induced by burst pacing stimulation in the T2DM group than in the control group (75% vs. 14.3%). Silencing or deleting the essential MAM gene GRP75 prevented tunicamycin (ERS inducer)-induced atrial remodeling and AF incidence (18.2% vs. 63.0%) [25].

In addition to ER-stress, oxidative stress is thought to contribute significantly to atrial remodeling and AF promotion. A previous study showed that two membrane subunits of nicotinamide adenine dinucleotide phosphate (NADPH) oxidase (p22phox and gp91phox) were significantly activated in the right atrial appendage (RAAs) tissue of AF patients and that these subunits played important roles in atrial reactive oxygen species (ROS) production and AF pathogenesis [28]. Meanwhile, superoxide dismutase (SOD), the most important antioxidant enzyme family that scavenges ROS, has been reported to be depleted in diabetic atrial tissue, thereby exacerbating the onset of AF [29]. It has been demonstrated that increasing the expression of mitochondrial HSP70 leads to an increase in ATP production and so regulates mitochondrial activity as well as ROS in cardiomyocytes. HSP70 stimulates mitochondrial SOD and inhibits the nuclear translocation of phosphorylated eukaryotic elongation factor 2 (eEF2) and apoptosis-inducing factor (AIF), resulting in improved mitochondrial function and decreased apoptosis in mice with left anterior descending artery ischemia/reperfusion [30,31]. Nobuyuki Murakoshi et.al. investigated the therapeutic benefits of antioxidants and HSP70 on age-related AF. In older rats (9-month-old), AF was induced 17 times out of 18 stimulation procedures (94.4%), compared to 15 times out of 18 stimulation procedures (83.3%) in younger animals (3-month-old). It has been reported that the peroxisome proliferator-activated receptor gamma (PPAR-γ) induces enzymes involved in ROS scavenging. Pioglitazone, a PPAR-γ activator, significantly reduced AF duration in aged rats to 45.6 ± 10.5 seconds (young, 30.7 ± 7.3 second; aged, 107.4 ± 24.2 second) [32]. Meanwhile, PPAR-γ activator supplementation has been shown to increase HSPA1 mRNA and protein levels in the atria of an AF rat model, inhibit age-related arrhythmogenic atrial remodeling, and AF perpetuation by improving antioxidant capacity and inhibiting the mitochondrial apoptotic signaling pathway [32]. It should be noted that PPAR-γ activators and HSP70 could be novel upstream therapies for AF.

**Figure 1:**
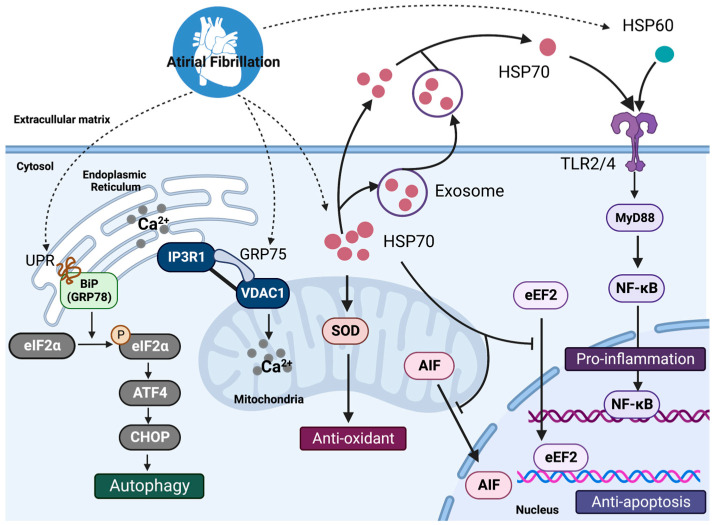
The mechanisms regulated by HSPs in atrial fibrillation. Intracellular HSP70s and HSP60 participate in autophagy, ER-stress, oxidative stress reaction, thereby exerting antioxidative and antiapoptotic effects and preventing atrial remodeling. However, when released into the extracellular matrix in free state, HSP60 and HSP70 bind to TLRs on the surface of the membrane to induce inflammatory responses by activating the MyD88/NF-κB pathway. HSP, heat shock protein; UPR, unfolded protein response; GRP78, glucose-regulated protein 78; eIF2α, α-subunit of eukaryotic initiation factor 2; ATF4, activating transcription factors 4; CHOP, CCAAT/enhancer-binding protein homology protein; IP3R1, inositol 1,4,5-trisphosphate receptors; VDAC1, voltage-dependent anion channel-1; SOD, superoxide dismutase; AIF, apoptosis-inducing factor; eEF2, eukaryotic elongation factor 2, NF-κB, nuclear factor kappa-B, TLR4, Toll-like receptors 4.

Similar to HSP60, an immediate release of HSP70 into the circulation and a modulation of TLR2 and TLR4 on monocytes after coronary artery bypass surgery (CABG) have been observed [33]. TLR2 levels were elevated in both monocytes and atrial tissue of patients with AF, and elevated atrial levels of TLR4 have been seen in patients with AF and heart failure [34,35,36]. Increased extracellular HSP70 increased the production of proinflammatory mediators (tumor necrosis factor-α [TNF-α] and interleukin-6 [IL-6]) in a CD14-dependent manner via the TLR2/4-mediated MyD88/NF-κB pathway [37,38]. HSP70 treatment of cultured HL-1 cardiomyocytes enhanced the expression of intercellular adhesion molecule 1 (ICAM-1), IL-6, and keratinocyte-derived chemokine (KC) compared to controls [37]. TLR2 antibodies can prevent contractile dysfunction and cell death by reducing ICAM-1 expression in cardiomyocytes induced by HSP70. Intracellular HSP70s, on the other hand, have been shown to exhibit cardioprotective properties. In myocardial ischemia/reperfusion mice, Dillmann and colleagues found that knocking out HSP70 genes resulted in cardiac hypertrophy after myocardial ischemia/reperfusion injury, which may be related to several signaling pathways, including Jun N-terminal kinase (JNK), p38/mitogen-activated protein kinase (MAPK), Raf-1, and extracellular signal-regulated kinase (ERK) [39]. The anti-inflammatory impact of HSP70s in AF warrants further research (Figure 1).

### 3.3. Small HSPs and Atrial Fibrillation

Cardiomyocytes express large quantities of small HSPs, particularly HSP27, which localize to contractile proteins and the microtubule network, stabilizing the structure and preserving the contractile and electrophysiological properties of cardiomyocytes. Human HSPB1 (Hsp27) levels in RAAs and LAAs tissue samples are reduced in the severe stage of persistent AF [9]. Overexpression of HSPB1, as well as HSPB6, HSPB7, HSPB8 can independently play a protective role against tachypacing-induced Ca^2+^ transient reduction via reducing the formation of F-actin stress fibers [40]. Brundel et al. also demonstrated that phosphorylated HSPB1 (HSP27) could prevent the atrial tachycardia–induced Ca^2+^ handling, L-type Ca^2+^ channel current reduction and associated action potential duration (APD) shortening in tachypaced HL-1 cardiomyocytes and isolated canine atrial cardiomyocytes [41]. Similar protective effects were observed when overexpressing HSP27, which accelerates the recovery of structural damage in AF. HSP27 may shield the contractile proteins from AF-induced cleavage by cysteine proteases (such as calpain 1) [8,42]. GGA-59 treatment of HL-1 cardiomyocytes accelerated recovery from the tachypacing-induced restoration of (acetylated) α-tubulin mRNA and protein levels, as well as cardiac troponin I and troponin T [43]. Several data suggest that HSPB1 protects the microtubule network, possibly through direct binding to histone deacetylases (HDAC) 6. HDAC6 emerges as a key regulator in AF progression by inducing α-tubulin deacetylation and, as a result, calpain-induced microtubule disruption, and may thus be a druggable target in AF [44]. Tubastatin A and ricolinostat (ACY-1215), two powerful HDAC6 inhibitors, have shown therapeutic effects against microtubule disruption in mouse models of neurological disorders and cancer. Furthermore, tubastatin A protects against electric remodeling (L-type Ca^2+^ current reduction and APD shortening) and cellular Ca^2+^ handling/contractile dysfunction, as well as subsequent AF promotion, in a canine model of AF [45]. These findings suggest that HSPB1 protects the microtubule network, possibly by directly binding to HDAC6 and inhibiting its activity, preventing α-tubulin deacetylation, depolymerization, and subsequent degradation [43,46].

## 4. Clinical Application of HSPs in Atrial Fibrillation

Atrial Fibrillation (AF) typically progresses from a self-terminating paroxysmal condition to a chronic disease. AF can be divided into four categories according to duration, including paroxysmal AF, persistent AF, long-standing persistent AF, and permanent AF [47]. The rate of progression from paroxysmal to persistent or permanent AF varies between 8% and 22% after one year, but responsible mechanisms remain elusive [48,49]. Despite the high incidence, risk and rapid progression of AF, accurate diagnosis of AF can currently only be achieved by body surface electrocardiography (ECG) and Holter monitoring or other rhythm monitoring methods [50]. However, a body surface ECG can only be used to diagnose AF events, the severity and duration cannot be determined [51]. Current treatments for AF are in large part based on catheter ablation, which aims to eliminate either the trigger initiating AF or the underlying arrhythmogenic substrate using either heat (radio frequency ablation) or freezing (cryoablation). As a major risk factor for cardio-embolic stroke, AF multiplies the overall risk by five [52]. Therefore, it is critical to recognize and diagnose AF as early as possible, to forecast its progression and to define the stage of AF. Unfortunately, because of the persistent and progressive nature of cardiac arrhythmia, therapies for AF remain tough.

Biomarkers are widely used to predict and monitor many diseases, including cardiovascular diseases. There is a great need for biomarkers to stage AF and to improve the selection of the proper treatment for patients, and there is growing evidence that some heat shock proteins (HSPs) are important in predicting and staging AF. As previously mentioned, the HSP family includes a series of molecular chaperone proteins that are widely expressed in many tissue and organ species and has potential as a biomarker for the diagnosis and staging of AF, as reviewed in this article (Table 1).

### 4.1. HSP60 Predicts Atrial Fibrillation and Indicates the Stability of Sinus Rhythm

As a member of the HSP family, HSP60 is closely associated with the development of AF. Schäfler AE et al. discovered an increase in HSP60 protein in atrial myocardial samples from patients with chronic AF [17,53]. In the first study, they obtained RAAs from patients with sinus rhythmia (n = 6) or chronic AF (n = 8), respectively. After Western blot analysis, atrial myocardial HSP60 levels were 2.5-fold higher in patients with chronic AF than in those with sinus rhythm. In another experiment, they used a similar approach to analyze the expression levels of HSP60 as well as HSP60 co-protein HSP10 in the atrial myocardium of patients with chronic AF (n = 8) and sinus rhythm (n = 8), and similar to HSP60, the expression of HSP10 was increased 2.4-fold in patients with chronic AF, and the above results were associated with high energy metabolism and high protein metabolism during the onset of AF. HSP60 plays a cardioprotective role during the onset of AF, and P. D. Neufer et al. also hypothesized that the increased level of HSP60 allows cardiac myocytes to better adapt to the high energy metabolic state during AF [54]. HSP60 has also been found to have a significant role in sinus rhythm stabilization in individuals with AF who undergo mitral valve surgery. The authors concluded that preoperatively low HSP60 levels were associated with postoperatively stable, spontaneously restored sinus rhythm [55].

Due to the limitations of test technology in the past, the results of peripheral blood tests were considerably different from those of local cardiac tissues. With the advancement of testing technology, HSP60 has been selected and studied by ^18^F-fluorodeoxyglucose (FDG) positron emission tomography (PET) imaging [56]. Researchers have found that HSP60 is associated with a reduced cardiac inflammatory response after radiofrequency catheter ablation (RFCA). ETA is a metabolically active organ producing inflammatory cytokines, which can diffuse into the adjacent atrial tissue and contribute to the genesis of AF. Researchers assess EAT activity by PET/CT. To evaluate the activity of EAT, tissue with Hounsfield units between -190 and -45 was defined as adipose tissue. Appropriate regions of interest (ROIs) were placed on the adipose tissue adjacent to the origin of the right coronary artery, and the maximum standardized uptake value (SUVmax) was recorded. Bi-Xi Chen et al. made the conclusion that HSP60 is associated with a reduction in myocardial inflammation after RFCA [57]. Among these inflammatory indicators, changes in HSP60 were closely related to changes in the activity of EAT before and after RFCA. The development of AF is intimately linked to the activity of the EAT, a metabolically active organ that may produce and release inflammatory substances, and it has even been demonstrated that the quantity of EAT in AF can be predicted independently [58]. Meanwhile, according to these studies, the level of HSP60 is independent of the maintenance of sinus rhythm and the duration of atrial fibrillation after RFCA.

### 4.2. HSP70 Predicts Atrial Fibrillation and Atrial Fibrillation Severity

HSP70 levels between AF patients and the healthy population did not differ significantly, according to a study by Jelena Kornej et al. The researchers included 67 patients with AF in this study and used an enzyme-linked immunosorbent assay (ELISA) to measure the circulating levels of HSP70 and HSP70 antibodies before and after catheter ablation, respectively. The analysis found no correlation between HSP70 levels and clinical presentation or ultrasound indices. In the AF cohort, patients with paroxysmal AF had lower HSP70 antibodies (median 43, IQR 28–62 μg/mL) at baseline compared to patients with persistent AF (53, 41–85 μg/mL, *p* = 0.035). Using uni- and multivariable regression analysis adjusted for age and gender, AF type was the only variable associated with HSP70 antibodies (Beta = 0.342, *p* = 0.008) [59]. Additionally, both HSP70 and its antibody levels were increased after catheter ablation treatment, and the increased levels were associated with the energy and duration of catheter ablation, as well as with the post-catheter ablation recurrence of AF [59]. Marion et al. conducted a prospective clinical trial that included 297 individuals, including 98 controls and 98 patients with AF scheduled for electrocardioversion (ECV) and 101 patients with atrial fibrillation scheduled for pulmonary vein isolation (PVI). The researchers followed the above population through outpatient visits and telephone follow-up, and obtained and analyzed their preoperative and postoperative blood samples at 3, 6, and 12 months. The researchers found that serum HSP70 levels, as well as HSP27 and HSP60 levels, did not differ significantly at different stages of AF. In addition, HSP70 did not show a correlation with recurrence after ECV or PVI [60]. Moreover, in patients undergoing coronary artery bypass surgery (CABG), HSP70 levels were also not significant in predicting the development of postoperative AF [15].

To investigate the protective effect of HSP70 on AF occurring under virous cardiomyopathies (CM), Too Jae Min and colleagues constructed models using SD rats. The CM models that they desired to study were ischemic cardiomyopathy (ICMP), the doxorubicin (DOX)-induced non-ischemic dilated cardiomyopathy model, and pressure-overload hypertrophic CM model by transverse aortic constriction (TAC). Afterwards, hemodynamic parameters and left atrial HSP70 levels in experimental animals were measured. Too Jae Min et al. finally found that HSP70 expression was higher in all cardiomyopathy groups compared to controls and HSP70 levels were shown to be inversely linked with the induction rate of AF in the control group, but not in the CM group. Although this study did not further clarify the relationship between serum HSP70 levels and the development of AF in the CM population, it may provide new ideas for future studies and help to understand the protective role of HSPs in other cardiovascular diseases [61].

### 4.3. HSP70 and the Treatment of Atrial Fibrillation

Currently, warfarin and thrombin/factor X activated (FXa) direct inhibitors are used to prevent AF-related embolic episodes; however, all anticoagulants raise the risk of bleeding to varying degrees [62]. Approximately one-third of patients receiving anticoagulation also receive concurrent antiplatelet treatment, which raises the risk of bleeding [63]. Mikel Allende et al. demonstrated that induction of HSP70 expression using CM-695 can play an antithrombotic role without increasing the risk of bleeding by constructing a variety of murine thrombosis models. CM-695 induced increased HSPA1 expression and, in consequence, increased the amount of HSP70 in the murine aortic tissue [64]. Specifically, CM-695 significantly reduced the mortality rate in a lung thromboembolism model (*p* = 0.04), delayed thrombosis subsequent to Rose Bengal/laser light-induced carotid artery injury and delayed thrombosis subsequent to ferric chloride-induced injury of the carotid artery. This team also observed that, unlike the anticoagulants currently used, the anticoagulant effect of HSP70 was not accompanied with an increased risk of bleeding [64]. CM-695 may be potentially useful to treat patients who need to receive long-term anticoagulation [64]. HSP70 induction by TRC051384 or tubastatin A is a technique used to delay thrombus formation with little bleeding risk, and it is highly promising for treating AF patients and other conditions with a high risk of bleeding. HSP70 overexpression had no effect on hemostatic status or bleeding tendency [63,65]. They demonstrated in their research that HSP70, a product of HspA1A/B, may prevent thrombosis without raising the risk of bleeding through a gene expression-based strategy. The induction of HSP70 enhanced the expression of vascular thrombomodulatory proteins and increased circulating activated protein C upon thrombotic stimulus. As a result, pharmacological stimulation of HSP70 expression is a suitable technique for addressing the need for safe and consistent anticoagulation in patients with AF and other patients requiring long-term anticoagulation medication [64]. Meanwhile, HSP70 has been used in a variety of different cardiac illnesses in addition to being a target for the diagnosis and treatment of AF. For instance, HSP70 functions as a molecular chaperone that controls intercellular proteins and associated signaling pathways, including numerous transcription factors, enzymes, and autophagy-related proteins, in order to lower ROS, lower calcium overload, inhibit inflammatory reactions, and lower autophagy, which in turn lowers myocardial ischemia-reperfusion injury [39,66,67]. Intercellular HSP70 also reduces the inflammatory response to reduce cardiac ischemia-reperfusion injury, but intracellular HSP70 promotes the inflammatory response, and this discrepancy due to its distinct localization warrants additional exploration.

### 4.4. HSP90 in Atrial Fibrillation and Other Arrhythmogenic Diseases

In the light of our retrieval result, there is no report on the relationship between HSP90 and AF. Therefore, we make a conclusion that there is no definite relationship between HSP90 and AF according to the current research progress, and HSP90 cannot yet be used as a biological marker to assess the onset and progression of AF. However, it is reassuring to note that HSP90 has been shown to have an impact on a variety of cardiac arrhythmias, such as long QT interval syndrome, by affecting cellular ion channel-related proteins [68].

### 4.5. HSPB and the Diagnosis of Atrial Fibrillation

The family of HSPB (small HSP) consists of at least ten members, and they are expressed in various human tissues. Various HSPB members are highly expressed in the heart. HSPB1 is one of these members [69,70].

In a previous study by Brundel, B. J. et al., the changes in HSPB levels during AF and the protective effect of HSPB on AF were explored. The study included both cellular experiments and clinical trials. By studying atrial HL-1 cells, the researchers found that increased HSP27 expression protects against pacing-induced cardiomyolysis. Based on this finding, they did a clinical trial with 13 people with sinus rhythm, 14 patients with paroxysmal AF, and 17 patients with persistent AF. The clinical trial demonstrated that HSP27 expression was elevated in the atrial tissue of patients with AF and found that the level of HSP27 expression was negatively correlated with the duration of the arrhythmia and the amount of rhabdomyolysis during paroxysmal and persistent AF [9].

Previous studies have shown that genetically inducing HSPB members has a protective effect against tachycardia-induced structural remodeling and contractile dysfunction [71]. Geranylgeranylacetone (GGA) is a common medication used to increase HSPB1 expression [72]. GGA was originally used as an anti-ulcer agent and is a non-toxic acyclic isoprenoid compound with a retinoid skeleton that induces HSPB1 synthesis in various tissues, including the gastric mucosa, intestine, liver, heart, retina, and the central nervous system [73,74]. GGA induces HSPB1 expression probably via the activation of heat shock transcription factor 1 (HSF1) [75]. The protective impact of GGA-induced HSPB1 expression on structural remodeling has been shown in experimental AF models, indicating that HSPB1 induction by GGA may have therapeutic utility [9]. Zhang. D et al. constructed a *Drosophila melanogaster* model to investigate the protective effects of HSPB1 and the HSP inducer GGA on atrial tachycardia. The different members of the *Drosophila melanogaster* small HSP family are located in the mitochondria, cytoplasm, and nucleus. Some of these proteins are functional direct homologs of human HSPs. Cardiomyocyte remodeling caused by tachycardia can be prevented in the *Drosophila melanogaster* model by GGA-induced HSP expression or by the single overexpression of DmHSP23, a homolog of human HSPB. This suggests a model and target for studying AF-related cardiomyocyte remodeling in humans [42]. Furthermore, GGA therapy protects against cardiomyocyte remodeling and, as a result, the incidence and recurrence of AF following cardioversion in canine models of atrial ischemia-related AF and tachypacing-induced AF promotion [76].

Besides GGA, L-glutamine, a semi-essential amino acid, also acts as an HSP inducer and can promote the expression of HSPs in various organs, especially HSP70 and HSP27 [77]. By promoting the cytoplasmic to nuclear translocation of triphosphorylated HSF1, L-glutamine enhances deoxyribonucleic acid (DNA) binding to heat shock elements, thereby increasing HSPs expression. The role of L-glutamine in the course of AF has been investigated, with serum HSPs levels decreasing considerably after 3 months of L-glutamine treatment, whereas HSP27 levels rebounded after 6 months [77]. Increased intracellular HSPs levels limit cell death, resulting in less pathogenic HSP release into the systemic circulation in cardiomyocytes. Higher baseline levels of HSP27 and HSP70 were associated with greater reductions in their serum levels after L-glutamine treatment. Some metabolites linked with carbohydrate, nucleotide, and amino acid metabolism were restored in patients with high HSP27 levels who received L-glutamine, implying that L-glutamine also plays a function in cellular metabolism [78]. However, atrial tissue was not obtained in this study, and the relationship between HSP concentration in cardiomyocytes and plasma HSP concentration remains to be further elucidated.

The above studies explored the changes in the levels of various subtypes of HSPs during AF and analyzed the relationship between the changes, occurrence, and progression of AF. Therefore, HSPs are expected to be used as a biological indicator for early AF diagnosis and as biomarkers for marking its progression and disease severity. Together, the induction of HSPs by GGA or other compounds is a promising new approach for the “upstream therapy” of AF without arrhythmogenic effects.

**Table 1:** Clinical Application of HSPs in atrial fibrillation.

Type	Family	Correlation with AF	Therapeutic Target	References
HSP70	HSPA	No significant difference between SR and AF.	Induced by TRC051384 or tubastatin A.	Kornej, J. et al. [59] Allende, M. et al. [64]
HSP27	HSPB	Lower baseline HSPB in patients with AF than in those with SR.	Induced by GGA or L-glutamine.	Brundel, B. J. et al. [9] Brundel, B. J. et al. [41] Zhang, D. et al. [42] Sakabe, M. et al. [76] Hamiel, C. R. et al. [78]
HSP60	HSPC	Higher HSP60 levels in patients with AF than in those with SR.	No treatment strategy for atrial fibrillation targeting HSP60.	Schafler, A. E. et al. [17] Schafler, A. E. et al. [53] Chen, B. X. et al. [57]
HSP90	HSPD	No significant difference between SR and AF.	No treatment strategy for atrial fibrillation targeting HSP90.	No reference.

## 5. Conclusions

Under pathological circumstances, heat shock proteins (HSPs) can be responsive to damage induced by stress to maintain proteostasis and prevent various forms of cardiomyocyte damage. Heat shock response (HSR) dysfunction is the root cause of proteostasis derailment brought on by atrial fibrillation (AF) and the ensuing electropathology. HSP60, HSP70, and small HSPs are the most studied protein members that are involved in the pathophysiology of AF. Intracellular HSPs participate in autophagy, ER-stress, oxidative stress, calcium homeostasis, and the PPAR-γ pathway, exerting antioxidant and antiapoptotic effects and preventing action potential duration (APD) shortening. However, when released into the extracellular matrix in a free state, HSP60 and HSP70 bind to TLRs on the surface of the membrane to induce inflammatory responses by activating the MyD88/NF-κB pathway. Thus, extracellular HSPs may have the potential to serve as diagnostic biomarkers of AF. In terms of clinical applications, on the one hand, there have been many studies on the association between the levels of HSPs and the development and progression of AF, which reflects the possibility of using HSPs as a biomarker in AF. HSP70, HSP27, HSP60, or their antibodies have shown diverse associations with the development and progression of AF. On the other hand, in the field of AF therapy, HSP inducers such as GGA, L-glutamine, and others, which can achieve the precise regulation of AF through upstream targets, have broad application prospects.
